# Supplementary material for: Collaborative intelligence in AI: Evaluating the performance of a council of AIs on the USMLE
Source: PLOS Digit Health. 2025 Oct 9;4(10):e0000787. doi: 10.1371/journal.pdig.0000787 (PMC12510544; doi:10.1371/journal.pdig.0000787)
Supplement: S2 File — (ZIP) [file pdig.0000787.s002.zip › S2 Data Raw data files containing unprocessed question inputs and LLM outputs including transcripts of deliberations/STEP3_Question-122-1_Type-MC-NJ.docx]

**Facilitator** **[Round 1]:** A 3-week-old male infant is brought to the office by his mother because of a 2-day history of white lesions in the mouth. The infant was born at term via uncomplicated spontaneous vaginal delivery; he has been breast-feeding well and is urinating and having bowel movements normally. At birth, he weighed 3289 g (7 lb 4 oz; 30th percentile). Today, he weighs 3629 kg (8 lb; 25th percentile). He appears adequately hydrated. Vital signs are normal. Physical examination discloses white patches on the tongue and buccal mucosa that do not come off when gently scraped with a tongue blade. Which of the following is the most appropriate pharmacotherapy? (A) Acyclovir (B) Fluconazole (C) Nystatin (D) Valacyclovir (E) No pharmacotherapy is necessary

**AI_D:** RESTATING THE QUESTION: This clinical scenario describes a 3-week-old male infant presenting with non-scrapable white patches on the tongue and inner cheeks. These signs are typical of oral thrush, a fungal infection usually caused by Candida species. The inquiry focuses on the best pharmacological treatment for this condition. PLANNING: The presented choices are Acyclovir, Fluconazole, Nystatin, Valacyclovir, and no pharmacotherapy. Each of these is associated with the treatment of different conditions. Acyclovir and Valacyclovir are antiviral medications typically used to manage herpes simplex virus infections. Fluconazole and Nystatin are antifungal agents. However, considering the patient's age and condition, the priority should be to select a safe and effective medication appropriate for a possible fungal infection in a neonate. CLINICAL REASONING: The white, non-scrapable lesions in this infant's mouth suggest oral thrush caused by Candida, a yeast-like fungus. A common cause of thrush in breastfeeding infants is the acquisition of Candida from the mother's vaginal flora during delivery or from the mother's nipples during breastfeeding. Acyclovir and Valacyclovir may not be suitable because they are antiviral medications used to treat viral infections, like herpes, not fungal infections. Fluconazole, an antifungal, is effective against Candida but is typically reserved for severe or refractory cases due to its systemic effects and potential side effects in infants. CONCLUDING: A suitable pharmacological choice for treating Candida infection in neonates would be a topical antifungal, which targets the organism causing the infection and has fewer systemic effects than an oral medication. Nystatin is a commonly used topical antifungal that adheres to the oral mucosa and changes the fungal cell's membrane permeability, resulting in cell death. RESPONSE: Given the patient's age, symptoms, and the need for safety and efficacy in treatment, the most appropriate pharmacotherapy in this case is Nystatin (choice C).

**AI_E:** 1) RESTATING THE QUESTION: The presented case involves a male infant who is 3 weeks old and has been suffering from the appearance of white lesions in his mouth for the past two days. He was born at term with a healthy birth weight and, despite a slight decline, currently still falls within the normal range for his age. He is being breast-fed, has normal excretion habits and looks adequately hydrated. During the physical examination, white areas are seen on his tongue and the inner cheeks of the mouth (buccal mucosa) that remain intact upon gentle scraping. The question is to identify the best pharmacological treatment for this infant's condition. 2) PLANNING: An assumption could be that the white patches in the infant's mouth might indicate an infection, specifically a fungal one usually caused by Candida species, known as oral thrush. Acyclovir and Valacyclovir, antiviral drugs primarily used for the treatment of herpes simplex virus, are less likely to be suitable as there are no mentions of vesicular lesions typical of herpes infections. Pharmacotherapy may not be necessary if the condition is benign and self-limiting. However, considering the symptoms and the infant's age, a fungal infection should be addressed. The next step would be to identify the best antifungal therapy, with Nystatin and Fluconazole being the main options. 3) CLINICAL REASONING: Oral thrush is often seen in infants and is caused by the fungus Candida. This infection commonly presents as persistent, white, cheesy patches in the mouth that do not come off when scraped. Nystatin and Fluconazole are both suitable treatments for oral candidiasis. However, while Fluconazole is effective, it is absorbed systemically and has potential side effects that might not be optimal for treatment of a newborn. On the other hand, Nystatin is not absorbed from the gastrointestinal tract and can be effectively used to treat superficial infections with minimal side effects. 4) CONCLUDING: Oral thrush, caused by Candida, is common among infants and presents with white patches that don't scrape off. While both Nystatin and Fluconazole are antifungal treatments, Nystatin is preferred for such a young patient due to its minimal side effects and localized action. 5) RESPONSE: The most suitable treatment would be (C) Nystatin. It is a safe and effective antifungal medication that is commonly used for treating oral candidiasis in infants.

**AI_B:** (1) RESTATING THE QUESTION: The case refers to a 3-week-old male infant, who shows signs of white lesions in his mouth for the past couple of days. He is well-hydrated, breastfeeding adequately, and has no growth abnormalities. The stand-out feature which seems to require attention is the presence of persistent white patches in his mouth that resist gentle scraping attempts. Given these symptoms, we need to determine the most effective medication from the given list, which includes Acyclovir, Fluconazole, Nystatin, Valacyclovir, or possibly no medication. (2) PLANNING: The white patches, that resist gentle scraping, suggest a possible yeast infection caused by Candida species, commonly called "thrush". We would normally associate acyclovir and valacyclovir with herpes simplex infections and we can rule out these options based on the type of lesions. Considering no pharmacotherapy would be appropriate if this was a case of benign oral vesicles which are self-limiting, but this seems unlikely given the persistence and specifics of the lesions. The choice must be made between fluconazole and nystatin, both known antifungal agents. (3) CLINICAL REASONING: Bearing in mind that both fluconazole and nystatin are antifungal medications effective against Candida species, which are the causative agents of thrush, it is essential to think about the age of the patient and the mode of drug administration. Given the infant's age, systemic antifungal treatment may be excessive and could potentially lead to adverse side effects. Nystatin, on the other hand, is a topical medication typically safe for use in infants. It is often applied as a liquid suspension that can be swabbed directly onto the white patches, thereby targeting the infected area specifically, and reducing potential systemic effects. (4) CONCLUDING: After considering the symptom, the infant's age and specifics of the lesion, it is most likely that the infant has oral thrush (candidiasis), a common condition in infants. For treating this, a topical antifungal like nystatin is a clinically appropriate choice owing to its efficacy against Candida and its relative safety for use in infants. (5) RESPONSE: The most appropriate pharmacotherapy for this infant would be (C) Nystatin.

**AI_C:** (1) RESTATING THE QUESTION: A 3-week old male infant has been brought in by his mother, who's concerned about white lesions that have appeared in his mouth over the past two days. The infant is breastfed, well-hydrated, and carries out his physiological functions normally. The infant was born healthily and his birth weight and current weight fall within a satisfactory percentile. Upon examining him, there are white patches both on his tongue and the buccal mucosa that don't scrape off easily. Given the situation, we have to determine the best pharmacotherapeutic course to take. The options include: - Acyclovir, an antiviral often used for herpes infections; - Fluconazole, commonly used to treat fungal infections like Candida; - Nystatin, another antifungal, often used for Candida infections in the mouth; - Valacyclovir, another antiviral used in herpes treatment; - Lastly, the possibility that no pharmacotherapy is necessary. (2) PLANNING: The white lesions are likely either a viral or a fungal infection. Herpes and Candida are primary considerations given the treatment options. Herpes could be ruled out due to the absence of painful sores or ulcers, which are common signs of it. Thus, it's more plausible to presume it's a fungal infection, which infants are susceptible to, especially if they are breastfed. (3) CLINICAL REASONING: Rotating this over through the clinical thought process, the infant's symptoms align with oral thrush, typically caused by the fungus Candida albicans. Oral thrush is common among infants, with white lesions being a distinctive characteristic. It is even more plausible in breastfed infants as the infection can transfer from a maternal vaginal yeast infection during delivery, or from nursing if the mother has a Candida infection on her nipples. Thus, an antifungal therapy would probably be the most effective. (4) CONCLUDING: Considering the infant's current health status, the presence of white lesions that are suggestive of Candida infection, and the lack of symptoms indicative of a viral infection, the most suitable therapeutic course would be to treat the presumed Candida infection. (5) RESPONSE: Thus, with everything taken into account, the best pharmacotherapy in this case would likely be (C) Nystatin, an antifungal medication usually used as a first-line treatment for candida infections such as oral thrush in infants.

**AI_A:** (1) RESTATING THE QUESTION: A 3-week-old term male infant is currently experiencing white lesions in his mouth that have been present for 2 days. The child has been feeding well and shows standard urinary and fecal output. He was born through a normal vaginal delivery and has no complications. His weight was in the 30th percentile at birth, and currently, it has dropped a bit to the 25th percentile. He seems to be in good health. An examination has uncovered white patches on his tongue and inside his cheeks which could not be removed by scraping. The question requires us to select the most suitable course of pharmacotherapy from the options provided: (A) Acyclovir, (B) Fluconazole, (C) Nystatin, (D) Valacyclovir, or (E) no pharmacotherapy. (2) PLANNING: In order to answer this question, we need to evaluate the symptoms described. The white patches that won't come off when scraped are indicative of a condition known as thrush, which is typically caused by the Candida species of fungi. Primarily, we need to consider antifungal agents for this condition. Acyclovir and Valacyclovir, options A and D respectively, are antiviral medications used primarily for treating infections caused by the herpes simplex virus, hence we can exclude these. Option E, 'No pharmacotherapy', can also be discarded given that the infant has a fungal infection which needs medication. We are thus left with Fluconazole (option B) and Nystatin (option C). Both of these drugs are antifungal medications, but their applications can be different. (3) CLINICAL REASONING: Fluconazole is commonly used for severe systemic infections such as candidemia, disseminated candidiasis, and infections of the central nervous system, while Nystatin is primarily used for superficial and mucocutaneous infections. Additionally, Fluconazole is known to have potential adverse effects in infants, including liver damage. As the affected infant shows no signs of systemic illness -only local infection- the most appropriate treatment would be Nystatin, which tends to be the first-line treatment for oral Candida infections in infants. (4) CONCLUDING: Given that the infant's symptoms align with a local oral fungal infection, the optimal pharmacotherapy involves using a drug that targets localized fungal growth without risking any systemic side effects associated with more potent antifungal agents. (5) RESPONSE: Hence, the most appropriate pharmacotherapy for the infant's condition would be Nystatin (Option C).

**Facilitator** The consensus among the AI responses is that the 3-week-old male infant's symptoms, specifically the presence of white lesions in his mouth that resist scraping, are indicative of oral thrush, a fungal infection typically caused by Candida species. This condition is common in infants and can be transferred from the mother during delivery or breastfeeding. The pharmacotherapy options provided include Acyclovir, Fluconazole, Nystatin, Valacyclovir, and the possibility of no pharmacotherapy. Acyclovir and Valacyclovir, both antiviral medications, are primarily used for treating herpes simplex virus infections and can be ruled out due to the absence of symptoms indicative of a viral infection. The option of no pharmacotherapy can also be discarded as the infant's condition requires treatment. The choice then lies between Fluconazole and Nystatin, both antifungal agents. Fluconazole is typically used for severe systemic infections and has potential adverse effects in infants, including liver damage. Nystatin, on the other hand, is primarily used for superficial and mucocutaneous infections and is considered safe for use in infants. Given the infant's age, the localized nature of the infection, and the need to avoid potential systemic side effects, the consensus is that the most appropriate pharmacotherapy for this infant's condition is Nystatin (Option C). This medication is a first-line treatment for oral Candida infections in infants due to its efficacy against Candida and its relative safety for use in infants.
